# *In vivo* Optogenetic Approach to Study Neuron-Oligodendroglia Interactions in Mouse Pups

**DOI:** 10.3389/fncel.2018.00477

**Published:** 2018-12-06

**Authors:** Domiziana Ortolani, Blandine Manot-Saillet, David Orduz, Fernando C. Ortiz, Maria Cecilia Angulo

**Affiliations:** ^1^INSERM U894, Institute of Psychiatry and Neuroscience of Paris, Paris, France; ^2^INSERM U1128, Paris, France; ^3^mechanisms of Myelin Formation and Repair Lab, Instituto de Ciencias Biomédicas, Facultad de Ciencias de la Salud, Universidad Autónoma de Chile, Santiago, Chile

**Keywords:** optogenetics, GABAergic interneuron, oligodendrocyte precursor cell, developing brain, somatosensory cortex, proliferation

## Abstract

Optogenetic and pharmacogenetic techniques have been effective to analyze the role of neuronal activity in controlling oligodendroglia lineage cells in behaving juvenile and adult mice. This kind of studies is also of high interest during early postnatal (PN) development since important changes in oligodendroglia dynamics occur during the first two PN weeks. Yet, neuronal manipulation is difficult to implement at an early age because high-level, specific protein expression is less reliable in neonatal mice. Here, we describe a protocol allowing for an optogenetic stimulation of neurons in awake mouse pups with the purpose of investigating the effect of neuronal activity on oligodendroglia dynamics during early PN stages. Since GABAergic interneurons contact oligodendrocyte precursor cells (OPCs) through *bona fide* synapses and maintain a close relationship with these progenitors during cortical development, we used this relevant example of neuron-oligodendroglia interaction to implement a proof-of-principle optogenetic approach. First, we tested Nkx2.1-Cre and Parvalbumin (PV)-Cre lines to drive the expression of the photosensitive ion channel channelrhodopsin-2 (ChR2) in subpopulations of interneurons at different developmental stages. By using patch-clamp recordings and photostimulation of ChR2-positive interneurons in acute somatosensory cortical slices, we analyzed the level of functional expression of ChR2 in these neurons. We found that ChR2 expression was insufficient in PV-Cre mouse at PN day 10 (PN10) and that this channel needs to be expressed from embryonic stages (as in the Nkx2.1-Cre line) to allow for a reliable photoactivation in mouse pups. Then, we implemented a stereotaxic surgery to place a mini-optic fiber at the cortical surface in order to photostimulate ChR2-positive interneurons at PN10. *In vivo* field potentials were recorded in Layer V to verify that photostimulation reaches deep cortical layers. Finally, we analyzed the effect of the photostimulation on the layer V oligodendroglia population by conventional immunostainings. Neither the total density nor a proliferative fraction of OPCs were affected by increasing interneuron activity *in vivo*, complementing previous findings showing the lack of effect of GABAergic synaptic activity on OPC proliferation. The methodology described here should provide a framework for future investigation of the role of early cellular interactions during PN brain maturation.

## Introduction

The advent of optogenetics (Boyden et al., 2005) and pharmacogenetics (Conklin et al., 2008; Dong et al., 2010) have allowed for a big progress revealing detailed mechanisms behind intercellular communication (Aston-Jones and Deisseroth, 2013; Adamantidis et al., 2015; Chen et al., 2018). Optogenetics is based on the expression of photosensitive protein-channels in specific cell types, making possible to activate or inhibit particular components of a given circuit by using light (Boyden et al., 2005; Han and Boyden, 2007; Aston-Jones and Deisseroth, 2013). Typically, the activation of a targeted neuron is achieved by photoactivation of a channel-rhodopsin 2 (ChR2) variant. The ChR2 is a cationic channel which activation leads to the depolarization of the cell membrane (Ernst et al., 2008). If the light-induced rise in the membrane potential reaches the activation threshold of intrinsic depolarizing ion channels expressed by the cell (i.e., voltage-dependent sodium or calcium channels), an action potential is triggered (Zhang et al., 2006, 2007; Ernst et al., 2008). By this mechanism, the generation of action potentials of targeted neurons can be controlled with high spatiotemporal precision.

While optogenetic techniques have largely contributed to our current understanding of neuronal network function in the mature central nervous system (CNS), their use to investigate the developing brain has been poor and only recent efforts have started to be done in this direction (Bitzenhofer et al., 2017a,b). A significant limitation when studying developing circuits is to reach enough levels of ChR2 expression on cell membranes in order to induce efficient responses by photostimulation. Given the variable protein expression during developmental stages, the heterologous expression of light-sensitive channels driven by endogenous cell promoters in the neonatal brain is not reliable enough (see Bitzenhofer et al., 2017a). In addition, *in vivo* brain photostimulation usually requires the placement of a ferrule or mini-optic fiber that is fixed on the cranial bone (Gradinaru et al., 2009; Yizhar et al., 2011), a procedure more difficult to apply on the soft skull of mouse pups. This issue has probably precluded the use of optogenetics to study developing circuits, privileging pharmacogenetics (Wong et al., 2018), a versatile but less precise technique in time and space.

During early postnatal (PN) development, neurons form transient circuits before establishing mature networks (Anastasiades et al., 2016). Interestingly, oligodendrocyte precursor cells (OPCs), the major source of myelinating oligodendrocytes in the CNS, receive a transient synaptic input from GABAergic interneurons during early PN development (Vélez-Fort et al., 2010; Zonouzi et al., 2015). Indeed, OPCs are the only non-neuronal cells synaptically contacted by neurons in the CNS (Bergles et al., 2000). In the somatosensory (barrel) cortex, the interneuron-OPC connectivity reaches a peak at PN10, 1 day prior to oligodendrocyte (OL) differentiation, and then declines progressively to disappear during the fourth PN week (Vélez-Fort et al., 2010; Balia et al., 2015; Orduz et al., 2015). Although the genetic inactivation of a specific interneuron-OPC synapse does not impair the proliferation and differentiation of OPCs at early PN stages (Balia et al., 2017), these data indicate the existence of a close relationship between interneurons and OPCs during a critical period for cortical circuit construction. Interneuron activity may thus affect OPC function through different synaptic and extrasynaptic mechanisms in the immature neocortex (Maldonado and Angulo, 2015). Indeed, in addition to interneuron-OPC synaptic interactions, OPCs express extrasynaptic GABA_A_ receptors (Passlick et al., 2013; Balia et al., 2015). Moreover, interneurons directly communicate with OPCs in the developing brain by secreting over 50 paracrine factors such as fractalkine that promote OPC differentiation (Voronova et al., 2017).

In this article, we describe an experimental protocol to activate cortical GABAergic interneurons *in vivo* by using optogenetics in mouse pups and analyze the effect of interneuron activity in OPC dynamics. We tested Nkx2.1-Cre and Parvalbumin (PV)-Cre lines to drive the expression of ChR2 in subpopulations of interneurons. We analyzed the functional expression of this photosensitive channel by combining patch-clamp recordings with photostimulation in acute somatosensory cortical slices. We found that ChR2 expression was insufficient in the PV-Cre mouse line at PN10, while the Nkx2.1-driven expression allowed for a reliable photoactivation in the developing cortex. Next, we developed a stereotaxic surgery to place a mini-optic fiber at the cortical surface to photostimulate ChR2-positive interneurons at PN10 in awake mouse pups. Finally, we analyzed the effect of interneuron photoactivation on the oligodendroglia population by conventional immunostainings. Our approach should provide a methodological tool to study the function of different neuron-oligodendroglia interactions in the early PN brain.

## Materials and Methods

### Transgenic Mice

All experiments followed European Union and institutional guidelines for the care and use of laboratory animals and were approved by French committees for animal care of the University Paris Descartes and the Ministry of National Education and Research (N°CEEA34.MCA.070.12). For experiments, we used transgenic Nkx2.1-Cre^(+/-)^:ChR2 (H134R)-YFP^(lox/+)^ and PV-Cre^(+/-)^:ChR2 (H134R)-YFP^(lox/lox)^ mice from PN8 to PN26 obtained after crossing from Nkx2.1-Cre (Kessaris et al., 2006), PV-Cre (JAX n°008069) and ChR2-lox (JAX n°012569).

### Electrophysiology and Photostimulation in Acute Slices

Acute parasagittal slices (300 μm) of the barrel cortex were obtained with an angle of 10° to the sagittal plane using a vibratome (Microm HM650V) as previously described (Vélez-Fort et al., 2010; Orduz et al., 2015). Slices were prepared in an ice-cold solution containing (in mM): 215 sucrose, 2.5 KCl, 1.25 NaH_2_PO_4_, 26 NaHCO_3_, 20 glucose, 5 pyruvate, 1 CaCl_2_, and 7 MgCl_2_ (95% O_2_, 5% CO_2_) and incubated for 30 min at 33°C in an extracellular solution containing (in mM): 126 NaCl, 2.5 KCl, 1.25 NaH2PO4, 20 glucose, 5 pyruvate, 2 CaCl_2_ and 1 MgCl_2_ (95% O_2_, 5% CO_2_). For recordings, slices were transferred to a recording chamber perfused with the same extracellular solution at 2–3 ml/min. An Olympus BX51 microscope equipped with a 40× fluorescent water-immersion objective, a Q-imaging camera and a CoolLed pE-2 fluorescent system (Scientifica, United Kingdom) allowed us to visualize the YFP fluorescent protein of ChR2-expressing interneurons in acute slices. Layer V ChR2-expressing interneurons were recorded at RT in whole-cell configuration with pipettes having a resistance of 3–5 MΩ and containing an intracellular solution with (in mM): 130 KGlu, 0.1 EGTA, 0.5 CaCl_2_, 2 MgCl_2_, 10 HEPES, 2 Na_2_-ATP, 0.2 Na-GTP and 10 Na_2_-phosphocreatine (pH≈7.3; 300 mOsm). Photostimulation of ChR2-expressing interneurons was obtained with an optic fiber (200 μm, NA = 0.66; Prizmatix Ltd., Israel) placed on layer V of the slice close to the patched cell and connected to a LED source delivering 460 nm wavelength light pulses (UHP-Mic-LED-460, Prizmatix Ltd., Israel). Local field potentials (LFPs) were recorded with a recording pipette filled with extracellular solution and located in layer V close to the optic fiber.

Whole-cell recordings were obtained using Multiclamp 700B, filtered at 3 kHz and digitized at 20 kHz. Digitized data were analyzed off-line using pClamp10.6 (Molecular Devices, United States) and Neuromatic package within IGOR Pro 6.0 environment (Wavemetrics, United States). The identity of interneurons was first assessed by analyzing the firing properties of neurons recorded in current-clamp mode as previously described (Orduz et al., 2015). Briefly, we used a depolarizing pulse of 800 ms to measure the instantaneous discharge frequency (F_initial_), the frequency at 200 ms (F_200_), the frequency at the end of the pulse (F_final,_) and the total frequency (F_total_). We calculated both early and late accommodations. The membrane input resistance R_m_ of neurons was calculated from a -200 pA hyperpolarized 800 ms step. The spike threshold, the first and second action potential amplitudes, and their corresponding durations were extracted from a 200 pA depolarizing pulse of 80 ms to calculate the amplitude reduction and duration increase. The amplitude of the after-hyperpolarization (AHP) was calculated as the difference between the threshold and the peak of the fast hyperpolarization.

Train pulses of light were used to elicit action potentials in ChR2-expressing interneurons (10 ms, 1 mW per pulse). Light trains of 10, 20, 30, and 50 Hz during 30 s were applied to define the optimal frequency inducing an effective activation of patched ChR2-expressing interneurons. For each frequency, we calculated the number of spikes (N_s_) with respect to the number of light pulses (N_LP_) and determined the percentage of success as [N_s_/N_LP_] × 100. LFPs were elicited by stimulating with light trains (10 Hz, 2 s). In a set of experiments, the extracellular solution contained: 50 μM (2R)-amino-5-phosphonovaleric acid (APV, ref: HB0225, Hellobio); 10 μM 2,3-dihydroxy-6-nitro-7-sulfamoyl-benzo[f]quinoxaline (NBQX, ref: ab120046, abcam) and 10 μM Gabazine (SR 95531, ref: ab120042, Abcam). Tetrodotoxin (TTX, ref: HB1035, Hellobio) was applied at a final concentration of 1 μM.

### Surgery and *in vivo* Photostimulation

**FIGURE 1:**
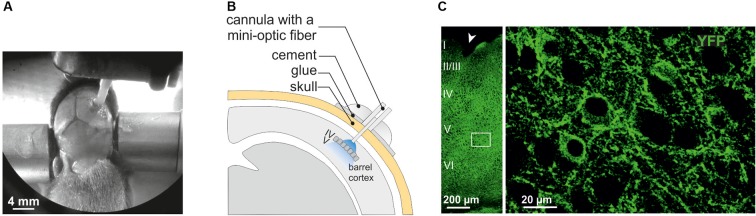
Surgery for *in vivo* optogenetic stimulation of interneurons of the somatosensory cortex in mouse pups. **(A)** An anesthetized PN10 mouse holding the implant and fixed in a stereotaxic apparatus is shown. Before to place the mini-optic fiber the skull is covered with two layers of glue. In this example, the skin of the animal was more widely incised for visibility on the picture. **(B)** Scheme of the mini-optic fiber implanted at the surface of the somatosensory barrel cortex. **(C)** Confocal images of ChR2-expressing interneurons in layers from I to VI (left; low magnification) and in layer V (right; high magnification of the white square in the left) of the barrel cortex of a Nkx2.1^Cre^:ChR2-YFP^Lox/+^ mouse at PN10. ChR2 expression driven by the Nkx2.1 promoter is detected by the YFP reporter (green). The arrowhead indicates a small lesion induced when removing the optic fiber of the pup after perfusion. It allows us to precisely locate the place of the mini-optic fiber.

Animals (PN10-11) were deeply anesthetized with ketamine/xylazine (0.1/0.01 mg/g, IP) and fixed in a stereotaxic frame adapted to mouse pups (Kopf Instruments, United States). The skin above the skull was disinfected and incised. In these young mice, the bone structure is not sufficiently transparent to let in the light into the cortex, and too soft to support the weight of the implant containing the mini-optic fiber. Thus, the skull was artificially stiffened with a double layer of glue before a hole was stereotaxically drilled above the barrel cortex area. For the adherence of the glue on the skull, the conjunctive tissue was first removed by a very local drop of HCl solution (1 mM). Caution was taken to apply this solution on a very restricted area to avoid any contact with the mouse skin or other tissues. Once cleaned, the skull was artificially stiffened with a double layer of glue (SuperGlue, Gel, ethyl-2-cyanoacrylate). The first layer was obtained by gently spreading out a first drop on the bone above the somatosensory cortex. After 10 min, a second layer of glue was similarly applied in the same region. Then, we waited around 20 min to ensure that the glue was completely dry before drilling the hole. Finally, the cannula accommodating the mini-optic fiber was implanted according to the following coordinates: 2.7 mm lateral to midline, 3.7 mm posterior to Bregma, and 0.1 mm depth from brain surface with an angle of 10° (Figures 1A,B; cannula: 1.25 mm; fiber: 200 μm diameter, NA = 0.66; Prizmatix Ltd., Israel). Some animals were implanted using an optrode containing a mini-optic fiber and a fine-wire recording electrode (Ni/Ag) of 50 μm diameter reaching layer V. In this case, a pin connector was fixed to the skull at the back of the brain to be used as a ground. Finally, the optogenetic implant was fixed to the skull with dental cement (Unifast Trad ivory 339104) and the skin was sutured around the implant (Mersilene^®^, EH7147H, Ethicon). Mice were recovered from anesthesia in an environment at 37°C before the photostimulation experiment. Once the animal was completely awake, the mini-optic fiber was connected to a 460 nm ultra-high power LED source through two optic fiber patch cords (UHP-mic-LED-460; Prizmatix Ltd., Israel). The first patch cord (200 μm diameter, NA 0.66) was connected from the mini-optic fiber to a rotary joint that spins freely, and the second (1000 μm, NA 0.66) was connected from the rotary joint to the optogenetics-LED optogenetics-LED source. This latter was connected to a current controller that in turn was commanded by a pulser device that creates programmable TTL pulses from a software (Prizmatix Pulser/Pulser PC Software, Prizmatix Ltd., Israel). Mice were photostimulated according to a 3 h protocol composed by 36 light trains of 30 s delivered at 10 Hz [10 ms on/90 ms off pulses; ∼3–4 mW per pulse, a power estimated from measurements in continuous wave (CW) mode] and separated by a resting period of 4.5 min. Considering that our duty cycle is 10%, we estimated that the total energy applied during a 30 s photostimulation train is equivalent to 9–12 mJ. Prior to the surgery, the power at the tip of each mini-optic fiber was systematically measured with an optical power meter (PM100D coupled with sensor S120C, Thorlabs, United States) by connecting it to the LED system operating in CW mode (maximum output power at the 200 μm mini-optic fiber tip: ∼4.5 mW). Once the surgery was completed, we set the values in the current controller to deliver the proper power at the tip of the fiber during the photostimulation. For LFP recordings, the recording electrode was connected to the headstage of a Multiclamp 700B amplifier through a 50 μm diameter Ni/Ag wire, and the ground of the mouse connected to the ground of the amplifier. Finally, the animal was placed inside a small (∼20 × 20 × 20 cm^3^) custom-made Faraday cage and light trains of 10 Hz were elicited to evoke LFPs in current-clamp (*I* = 0) voltage follower mode.

### Immunostainings, EdU Proliferation Assay and Countings

Fifteen minutes prior to photostimulation, intraperitoneal EdU injections were performed to labeled cells in the S phase of the cell cycle (10 mM EdU solution in PBS at 50 mg/kg; ref. C10340, ThermoFisher Scientific, United States). 1 h after the end of the photostimulation, mice were perfused with phosphate buffer saline (PBS) followed by 0.15 M phosphate buffer (PB) containing 4% paraformaldehyde (PFA, Electron Microscopy Sciences, United States), pH 7.4. Brains were then left in PFA for 2 h at 4°C before washing and storing them in PBS at 4°C. The day of immunostainings, coronal vibratome slices (100 μm) were prepared in PBS at 4°C and permeabilized with 1% Triton and 4% Normal Goat Serum (NGS) during 2 h at RT under agitation. Then, slices were first incubated at 4°C under gently agitation for 3 nights with rabbit anti-Olig2 (1:400, ref. AB9610, Merck-Millipore) and mouse anti-CC1 (1:100, ref. OP80, Calbiochem) diluted in 0.2% Triton X-100 and 2% NGS and revealed for 2 h at RT with secondary antibodies coupled to Alexa-405 and Alexa-633, respectively (1:500, ThermoFisher Scientific). EdU was revealed for 2 h at RT after immunostainings by using the Clik-iT EdU Alexa-555 (C10638, ThermoFisher Scientific). Between each incubation step and at the end of the protocol, slices were rinsed 3 times in PBS for 10 min under genlty agitation.

Confocal images (0.75 μm z-step) were acquired using a 63X oil objective (NA = 1.4) with a LSM 710 confocal microscope (Zeiss) and processed using NIH ImageJ^1^ as described (Balia et al., 2017). Cell densities were obtained by counting Olig2^+^, CC1^+^, and EdU^+^ cells with the ROI manager tool (ImageJ) and by dividing the number of cells by the volume, as previously described (Balia et al., 2017).

### Statistics

All data were expressed in mean ± SEM. A level of *p* < 0.05 was used to designate significant differences. Two group comparisons were performed using the non-parametric Mann–Whitney Test. Multiple comparisons were done with the non-parametric Kruskal–Wallis Test followed by a Dunn’s multiple comparison test. Statistics and plotting were performed using GraphPad Prism 5.00 (GraphPad Software Inc., United States).

## Results

### Functional ChR2 Expression in Cortical GABAergic Interneurons of Cre-Lox Transgenic Mice in Brain Slices

Three major cortical GABAergic interneuron subtypes can be defined according to the expression of three different markers: PV-, somatostatin (SST)-, and ionotropic serotonin receptor 5HT3a (5HT3aR) (Rudy et al., 2011). PV and SST interneurons constitute about 70% of the total population whereas 5HT3aR interneurons about 30% (Rudy et al., 2011). Cortical GABAergic interneurons are mainly born in the medial and caudal ganglionic eminences (MGE and CGE, respectively). The MGE is the site of origin of 60% of the cortical interneurons, mostly PV- and SST-interneurons (Marín, 2013; Wamsley and Fishell, 2017), while the CGE is the second source producing approximatively 30% of interneurons (Anderson et al., 2001; Nery et al., 2002). More recently, Gelman et al. (2011) also demonstrated that the embryonic preoptic area (POA) is a third source and suggested that this region contributes with approximately 10% of all GABAergic interneurons in the murine cerebral cortex. POA-derived progenitors give rise to PV, SST, and Reelin-expressing interneurons (Marín, 2013).

The specification of cortical interneurons in each generating area depends on a transcriptional network that regulates interneuron development. Among transcription factors important for interneuron specification, Nkx2.1 is specifically expressed in MGE- and POA-derived progenitors that generate 70% of cortical interneurons. These progenitors mainly generate both PV and SST interneurons with those in the POA producing more heterogeneous neuron subtypes (Marín, 2013; Wamsley and Fishell, 2017). Interestingly, the earliest wave of OPCs is also generated from Nkx2.1-expressing progenitors settled in the MGE and POA (Kessaris et al., 2006). Nevertheless, most of the Nkx2.1-derived precursors giving rise to oligodendroglia are eliminated by PN10 and thus should not significantly interfere to the ChR2 targeting of Nkx2.1-derived interneurons from this PN stage (Kessaris et al., 2006).

**FIGURE 2:**
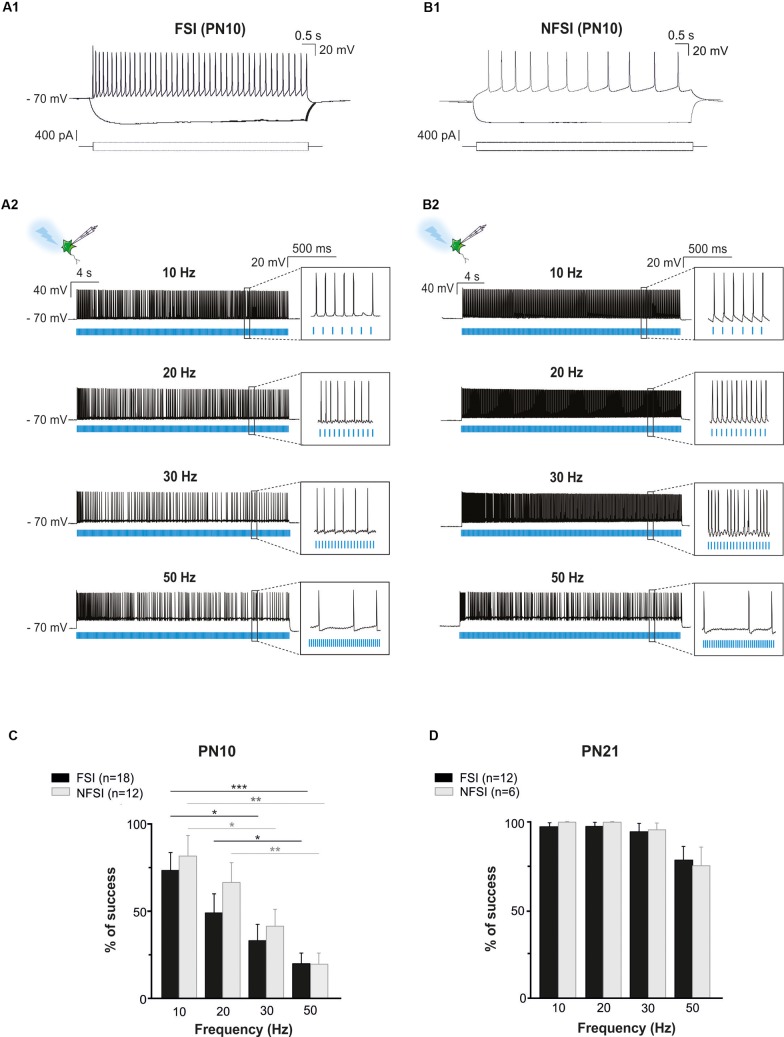
Photoactivation of ChR2-expressing FSI and NFSI in PN10 Nkx2.1^Cre^:ChR2-YFP^Lox/+^ mouse pups in brain slices. **(A1,B1)** Current-clamp recordings of a FSI **(A1)** and a NFSI **(B1)** expressing ChR2 in cortical layer V at PN10. Note the differences of firing properties between the two cells in response to 800 ms depolarizing and hyperpolarizing steps (bottom square pulses). **(A2,B2)** The same FSI **(A2)** and NFSI **(B2)** were photostimulated (blue bars) with 10 ms light pulses during 30 s at different frequencies. **(C,D)** Average percentage of success to elicit action potentials with light trains at different photostimulation frequencies for FSI (black) and NFSI (gray) at PN10 **(C)** and PN21 **(D)**. ^∗^*p* < 0.05; ^∗∗^*p* < 0.01; and ^∗∗∗^*p* < 0.001 for comparisons across each interneuron subtype, Kruskal–Wallis Test followed by a Dunn’s *post hoc* Test; not significant differences (*p* > 0.05) between FSI and NFSI for each frequency are not indicated; Mann–Whitney test.

In the present study, we used Nkx2.1^Cre^:ChR2-YFP^Lox/+^ mice to drive the PN ChR2 expression in MGE- and POA-derived interneurons from embryonic stages (from ∼E11.5 which corresponds to the peak of interneuron production; Anderson et al., 2001). At PN10-12, YFP^+^ interneurons were easily detected in the Nkx2.1^Cre^:ChR2-YFP^Lox/+^ mouse line in all cortical layers (Figure 1C). We focused on layer V since our previous studies in interneuron-OPC interactions were performed in this layer (Figure 1C; Vélez-Fort et al., 2010; Balia et al., 2015, 2017; Orduz et al., 2015). We first examined the electrophysiological properties of YFP^+^ cells in layer V by using patch-clamp recordings in acute slices of the barrel cortex (Figures 2A1,B1 and Table 1). As expected from Nkx2.1-derived interneurons at this age, recorded YFP^+^ cells could be distinguished as fast-spiking interneurons (FSI; Figure 2A1) and non-fast-spiking interneurons (NFSI; Figure 2B1) by their characteristic action potential discharges in response to current injections (Daw et al., 2007; Orduz et al., 2015). FSI were mainly distinguished from NFSI by their pronounced AHP and a restricted spike duration increase and amplitude reduction during action potential discharges (Table 1; see Orduz et al., 2015). Other analyzed parameters such as the initial frequency, second spike duration and early and late accommodations were also different between the two groups (Table 1; see section “Materials and Methods”).

**Table 1:** Electrophysiological properties of FSI and NFSI at PN10 in Nkx2.1^Cre^:ChR2-YFP^Lox/+^ mice.

Parameter PN 10	FSI(*n* = 18)	NFSI (*n* = 12)	*p*-value	Comparison
F_total_ (Hz)	30.94 ± 2.84	31.67 ± 2.43	NS	–
**F_initial_ (Hz)**	**94.65 ± 8.43**	**131.20 ± 13.41**	**<0.05**	**FSI < NFSI**
F_200_ (Hz)	63.86 ± 5.13	68.06 ± 5.50	NS	–
F_final_ (Hz)	59.56 ± 5.66	54.01 ± 4.84	NS	–
**Early accommodation (%)**	**30.64 ± 2.03**	**44.87 ± 3.74**	**<0.01**	**FSI < NFSI**
**Late accommodation (%)**	**5.11 ± 1.55**	**11.12 ± 1.79**	**<0.05**	**FSI < NFSI**
Threshold (mV)	-39.86 ± 2.48	-35.88 ± 1.89	NS	–
First spike amplitude (mV)	74.23 ± 1.89	78.71 ± 1.60	NS	–
Second spike amplitude (mV)	73.30 ± 1.80	74.48 ± 1.62	NS	–
**Spike amplitude reduction (%)**	**1.02 ± 0.25**	**5.10 ± 0.91**	**<0.0001**	**FSI < NFSI**
First spike duration (ms)	1.32 ± 0.08	1.49 ± 0.16	NS	–
**Second spike duration (ms)**	**1.42 ± 0.08**	**1.89 ± 0.22**	**<0.05**	**FSI < NFSI**
**Spike duration increase (%)**	**7.84 ± 0.71**	**26.00 ± 2.62**	**<0.0001**	**FSI < NFSI**
**AHP (mV)**	**-11.07 ± 1.21**	**-5.63 ± 0.76**	**<0.01**	**FSI > NFSI**
**AHP width (ms)**	**30.17 ± 14.49**	**9.38 ± 1.96**	**<0.05**	**FSI > NFSI**
Rm(MΩ)	203.07 ± 16.62	206.74 ± 46.89	NS	


*F_*total*_, total frequency; F_*initial*_, instantaneous discharge frequency; F_*200*,_ frequency at 200 ms; F_*final*_, frequency at the end of the pulse; AHP, after-hyperpolarization; R_*m*_, membrane input resistance. Statistical differences are in Bold.*

To test whether ChR2 was functionally expressed at PN10 in Nkx2.1^Cre^:ChR2-YFP^Lox/+^ mice, we photostimulated recorded YFP^+^ interneurons using an optic fiber placed in Layer V. Independently of the interneuron identity, all tested YFP^+^ cells responded with action potential discharges to light-pulse trains (Figures 2A2,B2). While the success rate of the response during a 30 s-train delivered at 10 Hz was very high for both FSI and NFSI, a progressive decrease in the capacity of neurons to follow the stimulation train was observed when increasing the frequency from 20 to 50 Hz (Figures 2A2,B2,C). No significant differences were observed between FSI and NFSI for each tested frequency, indicating that it is not possible to set up specific parameters to exclusively activate either FSI or NFSI (Figure 2C).

**FIGURE 3:**
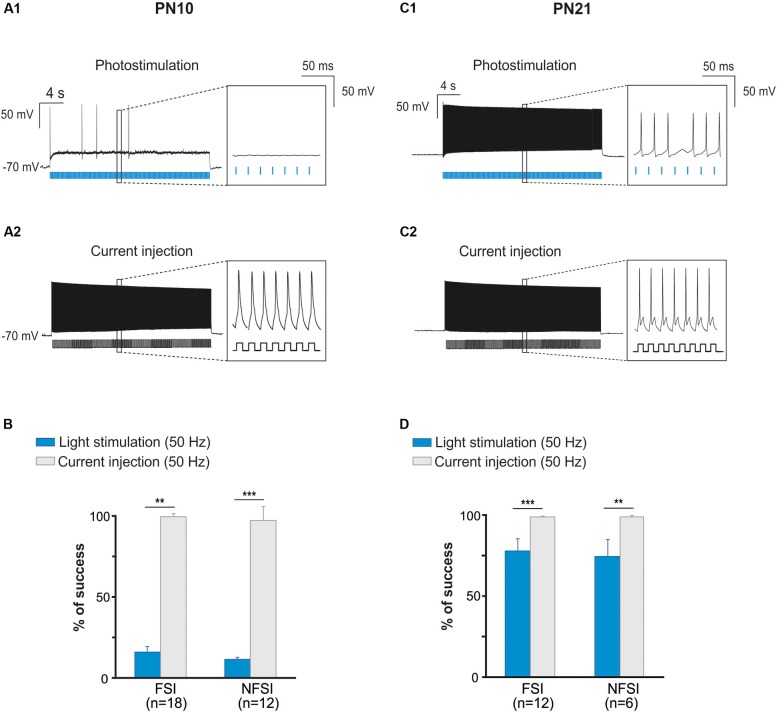
The fail of response at high frequency photostimulation in brain slices at PN10 is not due to interneuron immature membrane properties. **(A)** An identified ChR2-expressing interneuron recorded in current-clamp mode during a 50 Hz photostimulation protocol **(A1)** in a brain slice at P10. Note the numerous failures (inset) in response to blue light pulses (blue bars). The same neuron stimulated with current injections (400 pA, 10 ms; **(A2)**) at 50 Hz showed virtually no failures (inset). **(B)** Comparison between the percentage of success of photostimulation versus current stimulation at 50 Hz for FSI and NFSI in mice at PN10. **(C)** An identified ChR2-expressing interneuron recorded in current-clamp mode during a 50 Hz photostimulation protocol **(C1)** in a brain slice at PN21. Note the increased number of APs (inset) triggered by photoactivation (blue bars) compared with the interneuron at PN10 **(A1)**. The same neuron stimulated with current injections (400 pA, 10 ms; **C2**) at 50 Hz showed virtually no failures (insets). **(D)** Comparison between the percentage of success of photostimulation versus current stimulation at 50 Hz for FSI and NFSI in mice at PN21. ^∗∗^*p* < 0.01 and ^∗∗∗^*p* < 0.001, Mann–Whitney Test.

Two main reasons could explain the decreased response of YFP^+^ interneurons at higher frequency photostimulation trains: (1) a limited intrinsic capacity of immature interneurons to sustain high frequency discharges during long trains, and (2) a lower level of expression of ChR2 at PN10. To assess the first possibility, we mimicked the light train protocol with current pulse injections at 50 Hz for 30 s (Figures 3A1,A2). All recorded FSI and NFSI responded faithfully with action potentials to each current pulse injection, while the success rate during train photostimulation remains low at this early PN stage (Figures 3A,B). Therefore, the intrinsic properties of interneurons in younger mice are not responsible for a low success rate during higher frequency trains. To test for the level of expression of ChR2 during development, we performed the same experiments at PN19-22, i.e., during the third PN week (Supplementary Figure 1 and Table 2). Although we observed a maturation of the electrophysiological properties of interneurons during the second and third PN weeks (Tables 1, 2; Pangratz-Fuehrer and Hestrin, 2011), FSI were still distinguished from NFSI by the presence of a more pronounced AHP and small variations of the duration and amplitude spikes (Supplementary Figures 1A1,B1 and Table 2). Other parameters such as early accommodation and input resistance were significantly different between FSI and NFSI at a later developmental stage (Table 2). After recording the electrophysiological profile, we photostimulated the patched YFP^+^ interneuron using trains of photostimulation at 10, 20, 30, and 50 Hz (Supplementary Figures 1A2,B2). At PN19-22, the success rates for both FSI and NFSI were close to 100% for trains at 10, 20, and 30 Hz and dropped to around 80% at 50 Hz (Figure 2D and Supplementary Figures 1A2,B2), as expected from the limited capacity of ChR2 to follow high frequency trains (Lin et al., 2009). Indeed, current injections mimicking photostimulation at 50 Hz in PN19-22 mice caused a significantly higher success rate of ∼100% in FSI and NFSI (Figures 3C,D). Altogether, these results indicate that a lower level of ChR2 expression in the second PN week compared to 10 days later is probably at the origin of the decrease of the success rate with respect to the photostimulation frequency at PN10.

**Table 2:** Electrophysiological properties of FSI and NFSI at PN21 in Nkx2.1^Cre^:ChR2-YFP^Lox/+^ mice.

Parameter PN 21	FSI (*n* = 18)	NFSI (*n* = 8)	*p*-value	Comparison
F_totAl_ (Hz)	64.5 ± 6.42	64.38 ± 8.37	NS	–
F_initial_ (Hz)	185.7 ± 22.46	243.1 ± 24.14	NS	–
F_200_ (Hz)	132.9 ± 11.81	131.8 ± 20.54	NS	–
F_final_ (Hz)	126.5 ± 13.36	116 ± 16.79	NS	–
**Early accommodation (%)**	**21.99 ± 4.01**	**45.78 ± 6.61**	** <0.01**	**FS < NFS**
Late accommodation (%)	5.2 ± 1.93	6.49 ± 2.28	NS	–
Threshold (mV)	-44.26 ± 1.25	-44.59 ± 0.92	NS	–
First spike amplitude (mV)	77.84 ± 1.7	81.82 ± 1.57	NS	–
Second spike amplitude (mV)	76.23 ± 1.6	76.65 ± 1.6	NS	–
**Spike amplitude reduction (%)**	**2.0 ± 0.3**	**6.05 ± 3.68**	**<0.01**	**FS < NFS**
First spike duration (ms)	0.6 ± 0.04	0.77 ± 0.12	NS	–
Second spike duration (ms)	0.62 ± 0.04	0.87 ± 0.14	NS	–
**Spike duration increase (%)**	**3.47 ± 0.41**	**11.96 ± 1.98**	** <0.0001**	**FS < FS**
**AHP (mV)**	**-18.63 ± 0.93**	**-11.16 ± 1.73**	**<0.01**	**FS > NFS**
**AHP width (ms)**	**5.4 ± 0.77**	**2.29 ± 0.21**	**<0.05**	**FS > NFS**
**Rm(MΩ)**	**185.9 ± 14.55**	**242.8 ± 18.62**	**<0.05**	**FS < NFS**


*F_*total*_, total frequency; F_*initial*_, instantaneous discharge frequency; F_*200*,_ frequency at 200 ms; F_*final*,_ frequency at the end of the pulse; AHP, after hyperpolarization; R_*m*_, membrane input resistance. Statistical differences are in Bold.*

**FIGURE 4:**
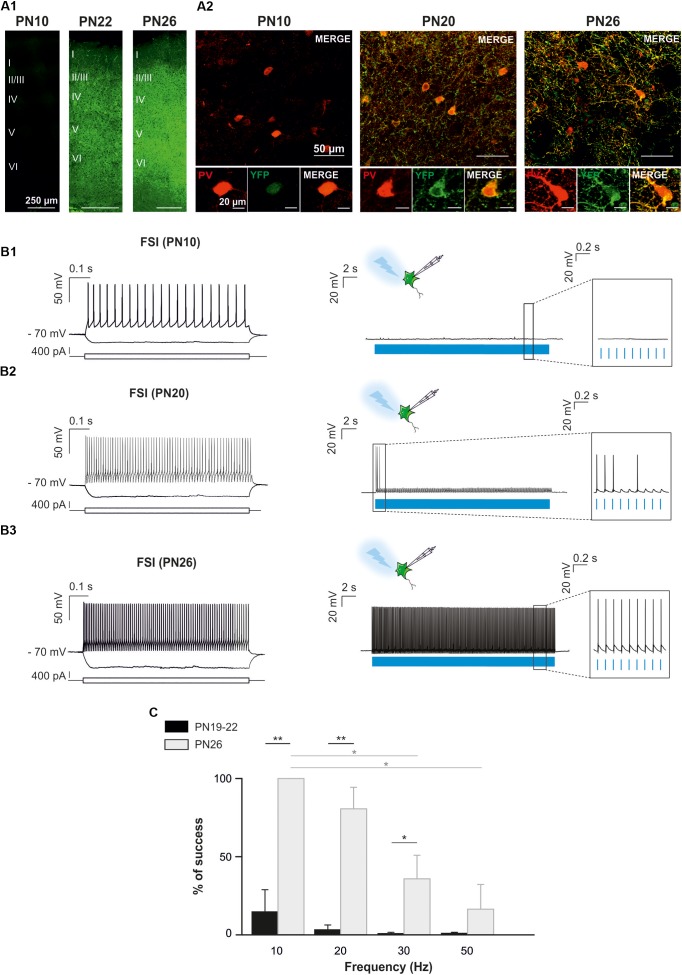
Optogenetic stimulation of FSI in PV^Cre^:ChR2-YFP^Lox/Lox^ mice during postnatal cortical development in brain slices. **(A)** Confocal images of cortical interneurons expressing ChR2 (YFP, green) and PV protein (red) at low (left) and high (right) magnifications at PN10, PN20 and PN26 mice. Note the faintly cytoplasmatic expression of YFP at PN10 compared to a brighter and more extensive membrane expression at later ages. **(B)** Current-clamp recordings of a layer V ChR2-expressing FSI **(B1)** at PN10. Depolarizing and hyperpolarizing steps are indicated (bottom square pulses). No action potentials were evoked by a 10 Hz photostimulation train (blue pulses) in this neuron (**B1** right, inset), but the photoactivation-induced response increased during development (**B2,B3**, right). Note that photoactivation (blue pulses) of a layer V ChR2- expressing FSI at PN26 evoked action potentials in response to every light pulse (B3 right, inset). **(C)** Average percentage of success to elicit action potentials with light trains delivered from 10 to 50 Hz at PN19-22 and PN26 in the PV^Cre^:ChR2-YFP^Lox/Lox^ mice. ^∗^*p* < 0.05; ^∗∗^*p* < 0.01, Kruskal–Wallis Test followed by a Dunn’s *post hoc* Test; not significant differences (*p* > 0.05) between FSI and NFSI for each frequency are not indicated.

Since PV-expressing FSI are highly connected to OPCs in the second PN week (Orduz et al., 2015), we also aimed at specifically photostimulating these interneurons in PV^Cre^:ChR2-YFP^Lox/Lox^ mice during this period. However, YFP^+^ interneurons were never detected in acute slices under the epifluorescence microscope and were very rarely observed under the confocal microscope at PN10 (Figure 4A). The scarcity of ChR2 expression in this mouse line at early PN stages was concomitant to the lack of response of FSI, recognized from their intrinsic electrophysiological properties, to a 10 Hz-photostimulation train at this age (*n* = 2, Figures 4B1,B2). Since this line has been extensively used in the adult (Cardin et al., 2010), we examined later developmental PN stages. Compared to PN10, immunostainings revealed an extensive co-labeling of PV and YFP in cell bodies and branches at PN20 and PN26 (Figure 4A). At PN19-22, 5 out of 7 neurons emitted at least one action potential in response to light train stimulation at 10 Hz (Figure 4B2) and one cell responded faithfully to each train pulse. In average, the success rate of discharge at 10 Hz was 14.62 ± 14.23% (Figure 4C). However, FSI rarely responded to higher frequency light trains at this age (Figure 4C). All recorded YFP^+^ neurons at PN26 responded faithfully with action potentials to each pulse of the light train stimulation at 10 Hz (success rate: 100%, *n* = 7, Figures 4B1,C2). Nevertheless, the success rate of YFP^+^ FSI progressively decreased at 20, 30, and 50 Hz-photostimulation trains (Figure 4). Therefore, there is an increased functional expression of ChR2 from PN10 to PN26 in PV^Cre^:ChR2-YFP^Lox/Lox^ mice. Considering that in Nkx2.1^Cre^:ChR2-YFP^Lox/+^ at PN21 the success rate of FSI is still high at 50 Hz (Figure 2C), a maximum level of ChR2 expression is probably not attained in PV^Cre^:ChR2-YFP^Lox/Lox^ mice in the fourth PN week.

In summary, contrary to a late PN expression of ChR2 in PV^Cre^:ChR2-YFP^Lox/Lox^ mice, ChR2 is already expressed in Nkx2.1^Cre^:ChR2-YFP^Lox/+^ mice at PN10, the age corresponding to the peak of synaptic connectivity between interneurons and OPCs in the somatosensory cortex (Orduz et al., 2015). Indeed, all recorded YFP^+^ interneurons responded to photostimulation trains at this developmental stage, although the success rate during a long train of 30 s significantly decreases at frequencies higher than 30 Hz. The most suitable light train frequencies to obtain a maximum and reliable number of action potentials during 30 s in Nkx2.1^Cre^:ChR2-YFP^Lox/+^ mice were between 10 and 20 Hz. Since FSI and NFSI fire at lower frequency rates in pups than in adults (Tables 1, 2), 10 Hz constitutes a good compromise to efficiently activate around 80% of interneurons during long light trains (30 s; Figure 2C).

### Light-Evoked Local Field Potentials in Acute Slices and *in vivo* in Nkx2.1^Cre^:ChR2-YFP^Lox/+^ Mice

**FIGURE 5:**
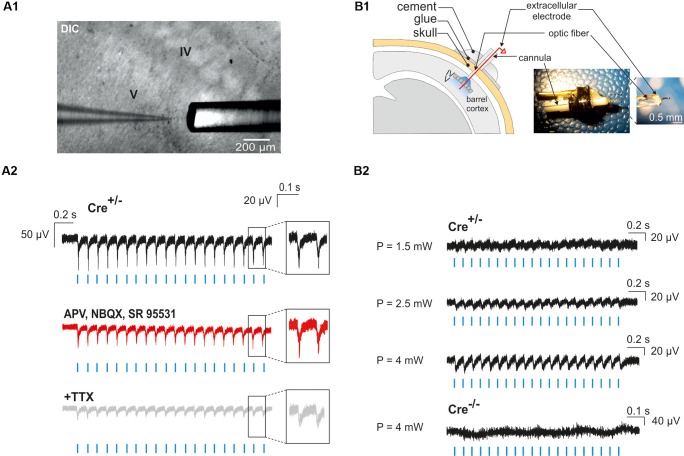
Photo-evoked LFPs in PN10 mouse pups in brain slices and *in vivo*. **(A)** Extracellular recordings were performed in the cortical layer V of brain slices during photostimulation with an optic fiber located just above the recording site **(A1)**. LFPs evoked by photostimulation (**A2**, blue lines) were faithfully reproduced in response to every light pulse (black trace, inset) in ChR2-expressing transgenic mice (Cre^+/-^). Recording of the same slice during bath application of glutamatergic and GABAergic antagonists (50 μM APV, 10 μM NBQX, 10 μM SR95531; **A2** red trace) and TTX (1 μM, **A2** gray trace) are shown. DIC: Differential interference contrast image. **(B)** *In vivo* extracellular recordings of cortical layer V were performed by the placement of a custom-made optrode containing a mini-optic fiber and a 50 μm Ni/Ag wire **(B1)**. *In vivo* LFPs were successfully evoked by photostimulation (**B2**, blue lines) above 1.5 mW of light power in a ChR2-expressing transgenic mouse (Cre^+/-^). The reported powers from 1.5 to 4 mW correspond to the power per pulse estimated from measurements in continuous wave (CW) mode. No responses were evoked in a Cre^-/-^ control mouse.

Neocortical GABAergic interneurons are inhibitory cells and constitute a minority of cortical neurons (10–20% in rodents; Rudy et al., 2011). For these two reasons, our observation of a single photoactivated interneuron does not necessarily ensure that light stimulation through the optic fiber will induce the activation of an interneuron population. To assess our ability to generate action potentials in a large number of ChR2-expressing interneurons, we first examined whether photostimulation elicited LFPs in acute cortical slices. We found that a 10 Hz photostimulation train induced LFPs with great fidelity in cortical layers II to VI (*n* = 2 slices in 2 animals, not shown), but since most of interneuron-OPC interactions in pups have been described in layer V (Vélez-Fort et al., 2010; Balia et al., 2015, 2017; Orduz et al., 2015), we analyzed in more detail the effect of interneuron activation in this layer (Figures 5A1,A2; *n* = 5 slices in 2 animals). These LFPs were less sensitive to the glutamatergic and GABAergic receptor antagonists APV, NBQX, and SR95531 than to the Na^+^-channel blocker tetrodotoxin (TTX) that abolishes action potential generation [Figures 5A1,A2; amplitude reduction of the first LFP: 4.32 ± 0.86% (*n* = 5) and 48.39 ± 12.1% (*n* = 3), respectively]. It is noteworthy that a TTX-insensitive component also persisted in all tested slices (Figure 5A2). It probably corresponded to the LFP generated by the current directly flowing through ChR2 channels in interneurons. These data indicate that recorded light-evoked LFPs upon 10 Hz photostimulation trains resulted from the direct generation of action potentials by multiple nearby ChR2-expressing interneurons instead of postsynaptic potentials (the latter could have been generated by inhibitory postsynaptic potentials or by disinhibition of inhibitory circuits resulting in increased excitatory postsynaptic potentials).

To analyze the effect of photostimulation *in vivo*, we performed a surgery at PN10 to implant an optrode including an optic fiber placed at the surface of the cortex. Since light poorly penetrates into scattering tissue, we tested whether neuronal responses were evoked by light in layer V by using a wire electrode to record light-evoked LFPs in this layer at a frequency of 10 or 20 Hz (Figure 5B1). To firmly maintain the optrode on the mouse skull, it was necessary to artificially harden the bone with a layer of glue before drilling (see section “Materials and Methods”). Once the animal was awake after surgery, it was placed in a chamber where the mini-optic fiber was connected to a 460 nm LED source through an optic fiber patch cord in order to record light-evoked LFPs (Figure 5B2; 4 Cre^(+/-)^ mice and 3 Cre^(-/-)^ mice). Photostimulation trains elicited weak responses when the power was set at 1.5 mW per pulse at the tip of the optrode (Figure 5B2). However, light-evoked LFPs increased for each light pulse at 2.5 and 4 mW (Figure 5B2). These responses were absent in Cre^(-/-)^ animals of the same littermates, used here as a control group (Figure 5B2).

Our results demonstrate that Nkx2.1^Cre^:ChR2-YFP^Lox/+^ mice can be used to activate a population of ChR2-expressing interneurons as early as PN10. We established the best conditions for whole-cell and LFP recordings in brain slices and set up the conditions to ensure layer V interneuron activation *in vivo*.

### Effect of *in vivo* Interneuron Activation on the Oligodendroglia Population

**FIGURE 6:**
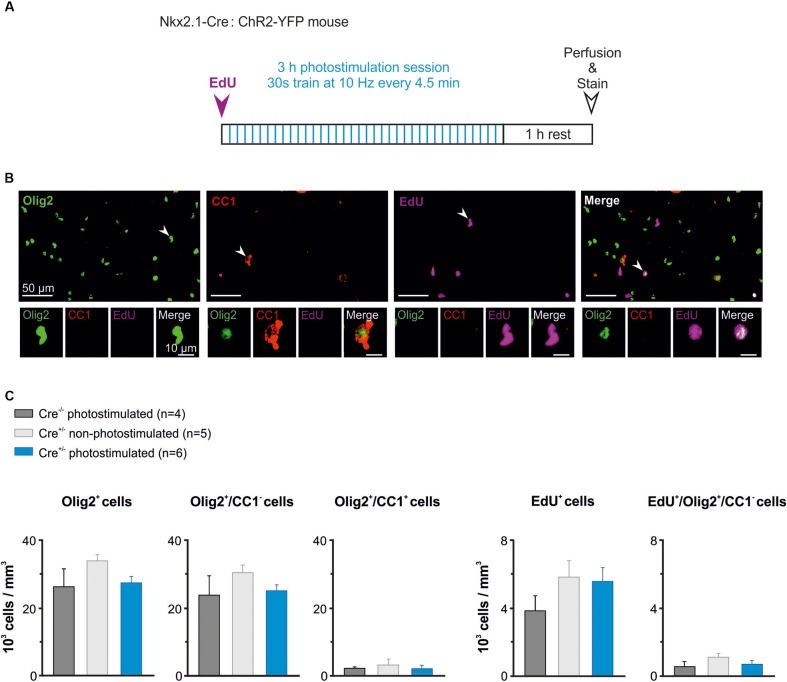
The *in vivo* photoactivation of layer V cortical interneurons at P10 does not modify oligodendroglia density. **(A)** Experimental design for a photostimulation protocol. Mice were injected with EdU (50 mg/kg) and then photostimulated during 3 h. **(B)** Olig2 (green), CC1 (red) and EdU (magenta) cells stained after the photostimulation session. An Olig2^+^/CC1 ^-^OPC is indicated in the first panel (arrowhead, insets). Only few Olig2^+^/CC1^+^ OLs were found at this age (arrowhead in second panel, insets). Note that not all EdU-stained cells were Olig2^+^ cells (arrowheads in the last two panels). **(C)** Density of total oligodendroglia (lig2^+^), OPCs (Olig2^+^/CC1^-^), differentiated OLs (Olig2^+^/CC1^+^), total proliferative cells (EdU^+^), and proliferative OPCs (Edu^+^/Olig2^+^/CC1^-^) in the three tested conditions. No significant differences were found for any comparison (*p* > 0.05, Kruskal–Wallis Test followed by a Dunn’s *post hoc* Test).

It was recently showed that photoactivation of layer V glutamatergic neurons has a rapid effect on increasing the proliferation rate of OPCs in juvenile mice (Gibson et al., 2014). Here, we analyzed whether the activity of GABAergic interneurons also influence OPC proliferation at PN10-11, when the synaptic connectivity between interneurons and OPCs is maximal in the somatosensory cortex (Orduz et al., 2015). We photostimulated awake pups to trigger a response of layer V ChR2-expressing interneurons using ∼3–4 mW per pulse (Figure 5B2). In these set of experiments, the wire electrode was not present on the implant (Figure 1B). Based on our previous characterization in slices, we chose 10 Hz as frequency for *in vivo* photostimulation. However, since 10 Hz represents a relatively low stimulation frequency for cortical interneurons that discharge at higher rates even in the immature mice (Table 1), we opted to multiply the number of stimulation trains. The photostimulation protocol lasted 3 h and comprised 36 stimulation trains of 30 s at 10 Hz (Figure 6A). Before the photostimulation session, we intraperitoneally injected 50 mg/kg of EdU, a thymidine analog that integrates the genome during the S phase of the cell cycle (Figure 6A). After the photostimulation session, we waited 1 h before perfusing the animal and removing the implant (Figure 6A). Then, immunostainings were performed to simultaneously identify all OL lineage cells (Olig2) and mature OLs (CC1), while EdU was revealed to detect proliferating cells. We considered Olig2^+^/CC1^-^ cells as OPCs and Olig2^+^/CC1^+^ cells as differentiated OLs (Figure 6B). We compared the effect of the photostimulation within three different experimental groups: 1) Cre^(-/-)^ ChR2-non-expressing photostimulated mice (*n* = 4); 2) Cre^(+/-)^ ChR2-expressing non-photostimulated mice (*n* = 5); and 3) Cre^(+/-)^ ChR2-expressing photostimulated mice (*n* = 6). The two first groups were considered as controls.

The region carrying the implant was recognized under the confocal microscope by the presence of a small lesion in upper layers of the cortex (Figure 1C). To ensure that we analyzed the photostimulated area, only the slices showing a small lesion were further imaged at high resolution in order to perform cell countings in layer V (1 to 3 slices per mice). Cell countings did not reveal any significant difference in the total density of Olig2^+^ cells, Olig2^+^/CC1^-^ OPCs or differentiated Olig2^+^/CC1^+^ OLs (Figure 6C, left). As expected for this early developmental age, most Olig2^+^ cells were OPCs in all conditions (Figure 6C, left). Moreover, we found that controls and photostimulated mice did not display any significant change in the total population of EdU^+^ cells or in the population of EdU^+^/Olig2^+^/CC1^-^ OPCs (Figure 6C, Right). It is noteworthy that no EdU^+^/Olig2^+^/CC1^+^ OLs were observed in our samples, suggesting more symmetric than asymmetric divisions of OPCs at PN10-11. In conclusion, we did not observe any difference in the density of proliferating OPCs after a 3 h stimulation protocol, suggesting a limited effect of interneuron activity on OPC proliferation at this age. This result supports and complements our previous findings showing that the GABAergic synaptic activity of OPCs does not play a role in oligodendrogenesis (Balia et al., 2017).

## Discussion

Here, we describe an experimental procedure to activate cortical GABAergic interneurons of PN10 mouse pups by using an optogenetic approach. We first determined that Nkx2.1-driven ChR2-expression allowed for a reproducible activation of cortical layer V GABAergic interneurons at PN10, whereas ChR2 was insufficiently expressed in the PV-Cre mouse at the same age. Combining photostimulation with both whole cell patch-clamp recordings in brain slices and *in vivo* extracellular recordings, we determined a suitable photostimulation protocol to ensure a reliable photoactivation of targeted cells. We also provide an experimental procedure to place a mini-optic fiber in the neonatal mouse brain in order to photoactivate interneurons in deep cortical layers of awake pups. Finally, we analyzed the effect of a particular neuronal photoactivation protocol on the oligodendroglia population in the neonatal neocortex.

Optogenetic studies have been proved to largely contribute to our current understanding of mature nervous system circuitry. However, only recent efforts have allowed for the implementation of this technique in the neonatal brain (Bitzenhofer et al., 2017a,b; Ahlbeck et al., 2018). Indeed, one of the major prerequisite for a selective and reliable optogenetic stimulation is to attain a sufficient level of opsin expression in targeted cells. This is more difficult to achieve in mouse pups due to the low or variable protein expression during early PN stages. In line with this, we found that the functional ChR2 expression of FSI in PV-Cre:ChR2-YFP^flox/flox^ mice was largely insufficient at PN10, preventing the emission of action potentials in these interneurons. PV, the major marker of FSI, starts to be expressed during the first PN week, but reaches high levels late during neocortical development. At PN10, PV mRNA expression revealed a moderate labeled of mRNA-positive cells in layer V of the somatosensory cortex compared to PN16 (De Lecea et al., 1995). A developmental increased pattern of PV expression was also observed in our immunostainings in concomitance with an increased ChR2 expression (Figure 4). Therefore, the PV-Cre transgenic mouse line is not appropriate for inducing a sufficient ChR2 expression driven by the PV promoter at PN10. Contrary to the late expression of ChR2 in PV-Cre:ChR2-YFP^flox/flox^ mice, this channel is more expressed at this age in Nkx2.1-Cre:ChR2-YFP^flox/+^ mice (Figure 1C). An efficient ChR2 expression was expected in this mouse line since Nkx2.1 is expressed in interneuron progenitors at early embryonic stages (Marín, 2013; Wamsley and Fishell, 2017). In addition, fate-mapping approaches used to track the development of Nkx2.1-derived interneurons exhibited the distribution of these neurons in the mouse somatosensory barrel cortex already in the first PN week (Butt et al., 2005). The choice of Cre transgenic mouse lines is therefore crucial for inducing a sufficient ChR2 expression early in PN development.

Another key aspect of an optogenetic approach is to ensure a reliable and consistent readout of action potential discharges triggered by photostimulation. This is particularly important considering recent reports indicating that, depending on the neuronal subtype and light stimulation parameters (i.e., pulse duration), photoactivation of ChR2 might silence instead of increasing neuronal activity (Lin et al., 2013; Herman et al., 2014). This undesirable effect is explained by a light-induced depolarization block that results from an overactivation of ChR2 and its subsequent exaggerated cation influx, leading to an insufficient repolarization (Lin et al., 2013; Herman et al., 2014). Importantly, Herman et al. (2014) reported that cortical interneurons were 2 to 4 times more susceptible to ChR2-dependent silencing than excitatory neuronal subtypes. Although the light pulse duration was the major responsible for the observed depolarization block in this study (Herman et al., 2014), a high frequency photoactivation can also be a triggering mechanism (Grossman et al., 2011). A first characterization of the response of ChR2-expressing neurons to photostimulation in brain slices is an important step, sometimes neglected, to define a pertinent protocol for *in vivo* experiments, even when behavioral tests are conducted.

In the present report, we determined that 10 ms light pulses delivered at 10–20 Hz reliably trigger action potential discharges during 30 s in ChR2-expressing interneurons of Nkx2.1-Cre:ChR2-YFP^flox/+^ mice at PN10. Interestingly, action potentials were triggered with high fidelity in these same cells at frequencies of 50 Hz upon depolarizing current injections. These results indicate that the inability of interneurons to respond to light stimuli is not due to their immature intrinsic membrane properties at this age, but likely to ChR2 expression levels and channel gated properties. Indeed, the opening and closing kinetics of ChR2 are slower than those of voltage-gated Na^+^ and K^+^ channels. Photocycle models for ChR2 describe two open states (O1–O2) and at least two non-conductive “D” sub-states characterized by a slow recovery –from 10 s to more than 20 s– after a dark period (Ernst et al., 2008; Stehfest and Hegemann, 2010; Grossman et al., 2011; Saita et al., 2018). This requirement of a long-lasting dark period to recruit ChR2 activation upon a new light stimulus partially explains the limited neuronal response to high frequency photostimulation (Ernst et al., 2008; Grossman et al., 2011). In addition, due to the O1-to-O2 turnover, short inter-pulse intervals preclude the proper ChR2 closing (Grossman et al., 2011; Saita et al., 2018). In this line, Grossman et al. (2011) found that in response to 50 Hz photostimulation trains applied to ChR2-transfected hippocampal neurons, only 63% of the responsive ChR2 were closed in the inter-pulse interval compared to 99% at 10 Hz, preventing membrane repolarization and leading to the depolarization block of these neurons (Grossman et al., 2011). Nevertheless, since not all the channels are activated upon a single photostimulation, a strong level of protein expression can partially compensate for ChR2 slow kinetics. Indeed, we observed that photostimulation of FSI delivered at 50 Hz in PV-Cre:ChR2-YFP^flox/flox^ mice in the fourth PN week was less efficient than that of FSI in Nkx2.1-Cre:ChR2-YFP^flox/+^ mice in the third PN week (∼15 vs. 75%, respectively).

Neuron-OPC communication has been extensively studied in both gray and white matters (Maldonado et al., 2011; Maldonado and Angulo, 2015). Interestingly, GABAergic synaptic contacts are established on OPCs early in the PN neocortex, reaching a peak at PN10 (Vélez-Fort et al., 2010; Balia et al., 2015; Orduz et al., 2015). In addition, interneurons are known to act in a paracrine manner by releasing fractalkine to promote OPC development in the immature neocortex (Voronova et al., 2017). Finally, Stedehouder et al. (2018) recently demonstrated that PV interneuron myelination by oligodendrocytes is an activity-dependent process, as shown for other neuronal cell types (Hines et al., 2015; Mensch et al., 2015; Wake et al., 2015). All these data indicate a close relationship between GABAergic interneurons and oligodendroglia during PN development, and suggest a role of the activity of these neurons in regulating oligodendroglia function. After determining the parameters for interneuron photoactivation *in vivo* at PN10, we examined the effect of an increased interneuron activity on OPC proliferation at this critical developmental stage for OPC development in the somatosensory cortex (Hill et al., 2014; Balia et al., 2017). We found that the activity of GABAergic interneurons did not change either OPC proliferation or the number of OPCs and OLs in response to a 3 h photostimulation session. This result is in line with our previous report showing no changes in OPC proliferation after the genetic inactivation of interneuron-OPC synapses at the same age (Balia et al., 2017). However, a previous study had shown that 30 min photoactivation of cortical glutamatergic neurons had a rapid effect on the proliferation of these progenitors in juvenile mice (Gibson et al., 2014). It is therefore possible that the activity of glutamatergic and GABAergic neurons in the neocortex play different roles in OPC function. Alternatively, other stimulation paradigms for interneuron activation, closer to their higher firing frequency, need to be tested. Indeed, OPCs might respond in a different manner to different stimulation paradigms (Nagy et al., 2017). To test this possibility, however, other variants of ChR2 would be probably more appropriate such as the form E123T/T159C that has a success rate higher than 80% at 60 Hz (Berndt et al., 2011), or the ChETA variant that allows for a stimulation up to 200 Hz (Gunaydin et al., 2010).

## Conclusion

In conclusion, this report provides a step-by-step description of an experimental protocol for the optogenetic interrogation of a neuron-oligodendroglia interaction in mouse pups. This methodology could be useful for instance to analyze whether neuronal activity promotes oligodendroglia migration by inspecting the cell density in different cortical layers after *in vivo* stimulation or to search for activity-dependent interneuron-OPC synaptic plasticity by performing electrophysiology in brain slices or immunostainings of synaptic compartments at the end of the *in vivo* protocol. It can also be adapted to other optogenetic tools and cellular interactions of the developing brain to get new insights on the role of glial cells *in vivo* during neuronal circuit formation and maturation.

## Author Contributions

The two co-first authors conducted optogenetic and electrophysiological experiments in brain slices and *in vivo* and performed data analysis. DO participated in initial experiments of the project. All authors wrote the manuscript. FCO and MCA designed experiments and supervised the project.

## Conflict of Interest Statement

The authors declare that the research was conducted in the absence of any commercial or financial relationships that could be construed as a potential conflict of interest.

## Footnotes

1 http://imagej.nih.gov/ij
