# Supplementary material for: In vivo Optogenetic Approach to Study Neuron-Oligodendroglia Interactions in Mouse Pups
Source: Front Cell Neurosci. 2018 Dec 6;12:477. doi: 10.3389/fncel.2018.00477 (PMC6291523; doi:10.3389/fncel.2018.00477)
Supplement: Supplementary file 1 [file Data_Sheet_1.PDF]

Supplementary Figure

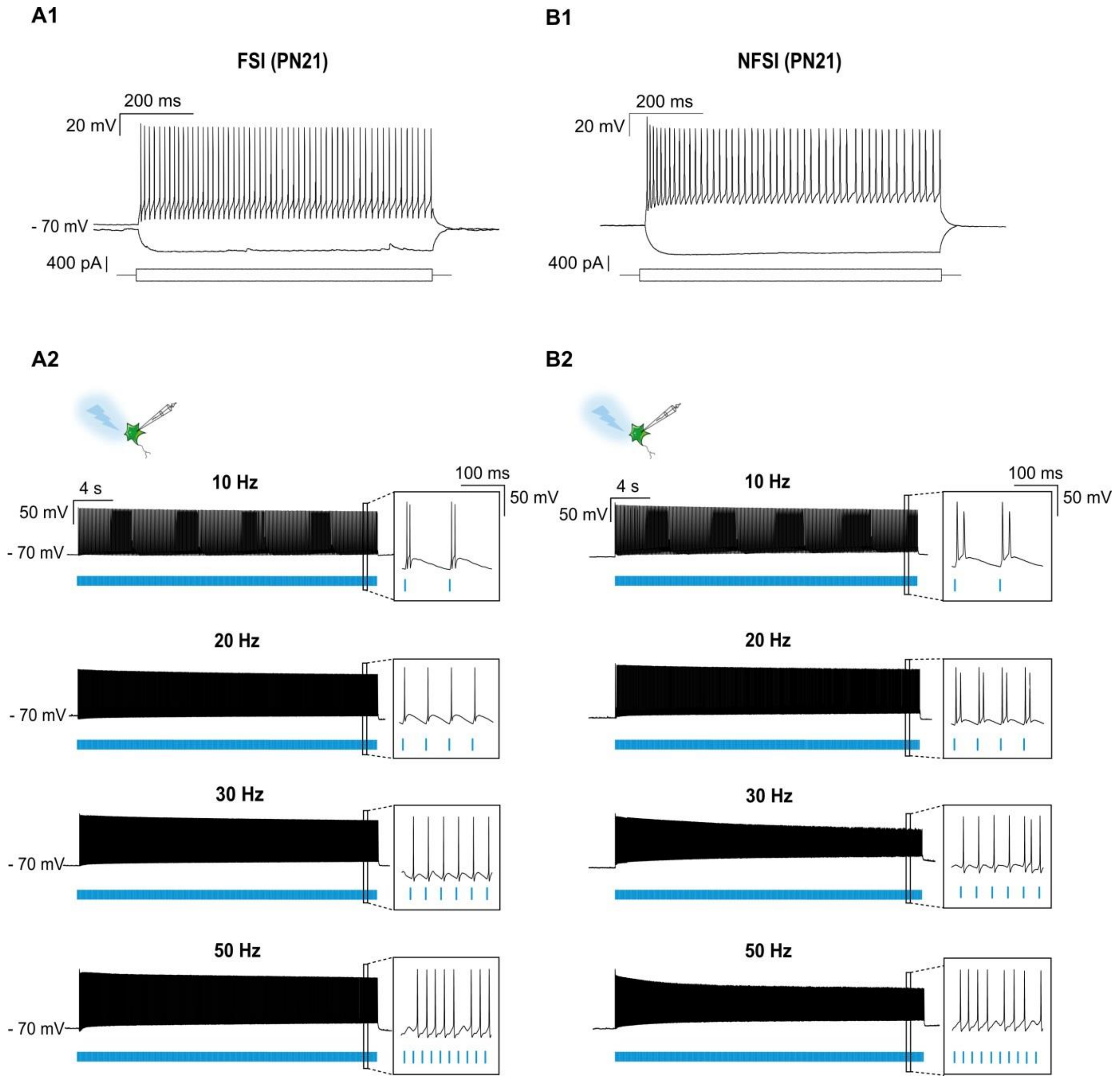

Supplementary Figure 1. Photoactivation of ChR2-expressing FSI and NFSI in PN21 Nkx2.1^{Cre}:ChR2-YFP$^{Lox/+}$ mouse pups in brain slices. A1 and B1. Current-clamp recordings of a FSI (A1) and a NFSI (B1) expressing ChR2 in cortical layer V at PN21. Note the differences of firing properties between the two cells in response to 800 ms depolarizing and hyperpolarizing steps (bottom square pulses). **A2 and B2**. The same FSI (A2) and NFSI (B2) were photostimulated (blue bars) with 10 ms light pulses during 30 s at different frequencies. Note that at this age there are virtually no failures (insets) in response to light pulses (blue bars) at any frequency of stimulation compared with P10 mice (see Figure 2).
